# Utilizing the Flexibility of Directional Deep Brain Stimulation Intraoperatively (if Needed) to Minimize Microelectrode Lead Repositioning

**DOI:** 10.7759/cureus.5276

**Published:** 2019-07-30

**Authors:** Ramsey Falconer, Tulsi Shah, Sean Rogers, Anna Green, Mahesh Shenai

**Affiliations:** 1 Neurology, Inova Neuroscience Institute, Falls Church, USA; 2 Neurology, Virginia Commonwealth University School of Medicine, Richmond, USA; 3 Neurosurgery, Inova Health System, Falls Church, USA

**Keywords:** current steering, microelectrode recording, axially asymmetric stimulation, segmented electrode, deep brain stimulation (dbs), directional deep brain stimulation, directional dbs, functional neurosurgery, parkinson's disease

## Abstract

This index case report describes the intraoperative use of an eight-contact directional deep brain stimulation (DBS) lead to avoid adjustment and repeat microelectrode passes after the initial pass elicited side-effects that suggested a slightly anteriorly placed lead.

While targeting the subthalamic nucleus (STN), intraoperative microelectrode recording (MER) confirmed that lead positioning and macrostimulation resulted in response at 1 mA but sustained dysarthria at 2 mA. This suggested a slightly anteriorly located electrode. The patient was becoming anxious, so instead of lead adjustment, an eight-contact directional DBS lead was placed to take advantage of the directional contacts, noting that a repeat pass could always then be performed. Segmented contact 11C showed symptom response at 0.5 mA and side-effect at 4 mA, resulting in a 3.5 mA therapeutic window.

Though no substitute for an accurately placed lead, this result suggests that the flexibility of directional stimulation could be considered in the intraoperative setting.

## Introduction

The field of deep brain stimulation (DBS) has fundamentally changed with the introduction of the directional DBS lead, which has allowed for the activation of tissue in an asymmetric fashion around the DBS lead, steering the current towards or away from intended or unintended pathways. This added flexibility of delivering energy that is axially asymmetric to the bore of the lead has potential benefit in the operating room during initial DBS implantation, one potential application of which is described here.

With the classic four-contact DBS electrode that lacked a directional DBS component, the stimulation and thus the volume tissue activation (VTA) was limited by being axially symmetric to the lead, thus delivering stimulation in relatively equal parts circumferentially around the lead. Programming the device was then pursued in an incremental level of complexity with the goal of expanding the VTA towards the intended target, without stimulating an unintended pathway to cause adverse effects. With the segmented eight-contact directional DBS lead, stimulation can be delivered in an axially asymmetric fashion, creating novel approaches to programming, improving therapeutic flexibility, and opening the door to other innovative applications.

In terms of outcomes, DBS has been shown to be at least equivalent to non-directional stimulation [1], and several advantages are starting to show, including the ability to utilize a larger therapeutic window between symptom response and side-effect. In addition, studies have shown that this wider therapeutic range can be achieved with a lower current threshold [1]. This can likely be attributed to both the smaller surface area of the directional contact creating a higher density of energy and thus a larger and more effective VTA for the power used, as well as the ability to deliver the energy in an axially asymmetric way towards the intended pathways and not towards those pathways whose activation would cause adverse effects. Additionally, these benefits are achievable while still maintaining the long-term symptom response and medication reduction expected from classic non-directional stimulation [2], without a trade-off of battery life or predicted device lifespan [3].

The potential advantage of directional stimulation stems from the fundamental purpose of DBS stimulation delivery: to shape a field of energy in such a way that activates the intended target without activating an unintended pathway. When the field is imprecisely shaped, adverse effects occur because the classic targets, such as the subthalamic nucleus (STN) for example, exist in a dense anatomic area with important adjacent structures that, when stimulated, can cause diplopia (oculomotor nucleus), paresthesia (medial lemniscus), mood changes (anterior STN), muscle contractions (internal capsule), gait changes (dentatorubrothalamic tracts), and more. Because of this narrow anatomical window, the accurate placement of the lead is paramount [4].

Microelectrode recording (MER) is used to precisely identify the surgical target for DBS and confirm a safe distance from nearby structures. Traditionally, MER involves having an awake patient perform various motor and speech tasks to functionally localize the effective target [5] while minimizing adverse effects producing changes in motor control or speech [6]. If initial positioning is suboptimal, the microelectrode is repositioned until minimal adverse effects and maximal relief of symptoms are achieved at the confirmed target, i.e. the largest therapeutic window. If an adverse effect is identified, the microelectrode is then adjusted by removal and reinsertion with a correction vector and can be repeated multiple times. Adjustment occurs based on the adverse effect and the source anatomic correlate, be it that the lead is too anterior, posterior, etc. When the ideal track is identified, the microelectrode is removed and replaced by the permanent DBS lead.

DBS is overall a relatively safe operation associated with a low risk of permanent neurological deficits (0.6% of implantations) [7]. The most severe adverse effect associated with DBS is intracerebral hemorrhage (ICH) [6]. Studies have indicated that symptomatic ICH occurs in approximately 2% to 3.7% of patients undergoing DBS [8-9]. Furthermore, it is estimated that the risk of ICH increases with each passing of the microelectrode by approximately 1.3% per passing [7,10-12]. Thus, reducing the number of microelectrode passes necessary to locate the intended target with the greatest therapeutic window may decrease the risk of ICH, subsequently reducing the permanent morbidities and mortalities associated with DBS.

Through this index case report, we describe a novel utilization of the eight-contact directional DBS lead to effectively avoid repeated intraoperative microelectrode passes when microelectrode stimulation response would otherwise have necessitated repositioning. While there is no substitute for an accurately placed lead, this case presented a situation where the limitations of a slightly anterior lead were negated by the directional contacts.

## Case presentation

The patient is a 65-year-old female with a history of symptom onset in the early 2000s, which began with a change in her right arm swing with walking and changes in her handwriting. Shortly thereafter, she developed a right-hand tremor at rest. She was given a diagnosis of Parkinson’s disease and started on carbidopa/levodopa, which resulted in significant improvement in her symptoms. Her symptoms then progressed over the following 10 years, and she was tried on varying combination of agents, all giving either sub-optimal response or side-effects. At the time DBS was discussed, she was taking one tablet of carbidopa/levodopa/entacapone 50/200/200 mg every two to three hours between six and seven times daily, and she was also using the Rotigotine patch at 8 mg daily. She was experiencing significant motor fluctuations and unexpected off spells.

**Figure 1:**
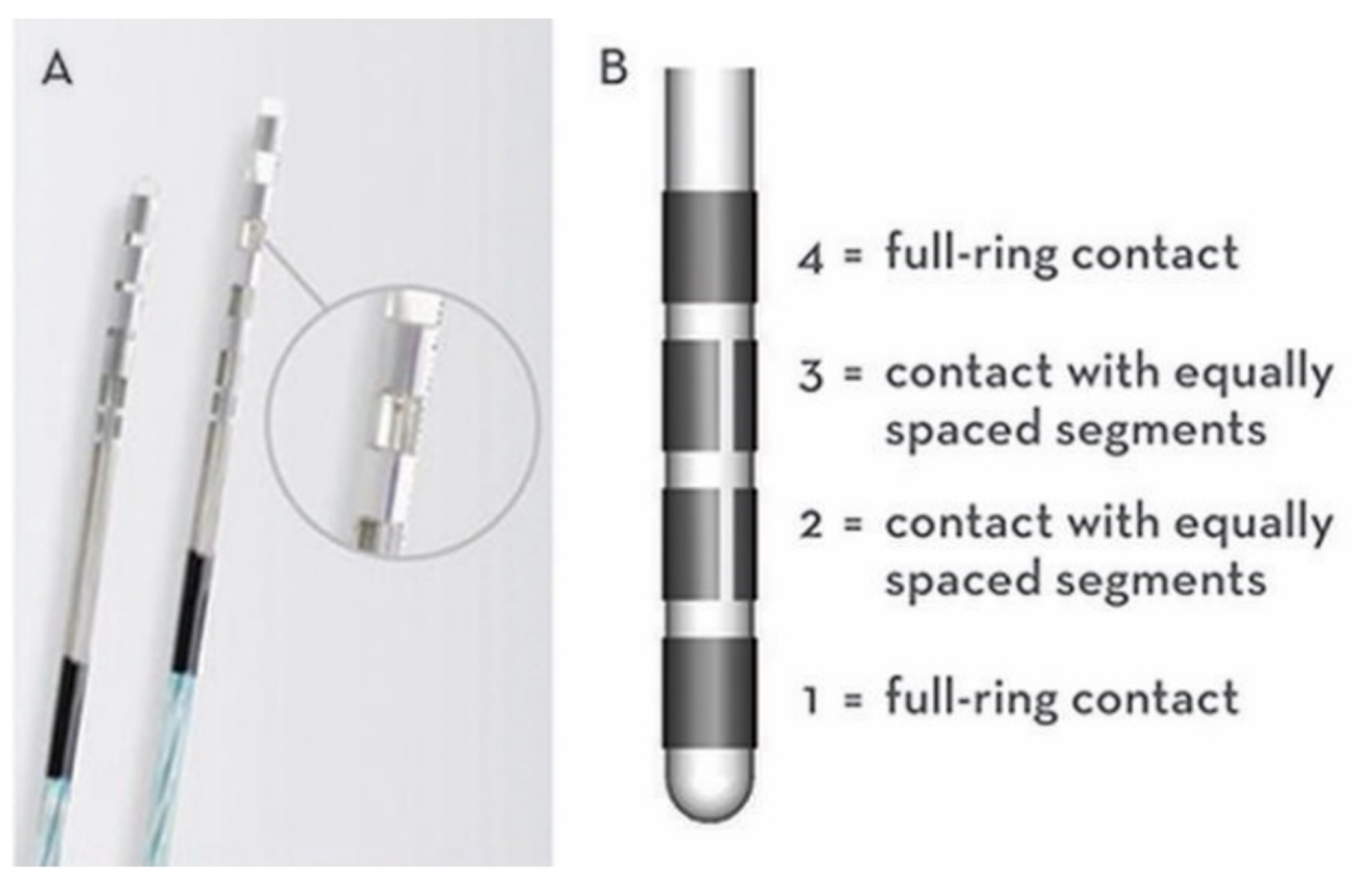
Abbott™ eight-segmented directional DBS lead showing the segmented middle two leads, each divided into three equally spaced contacts (~90 degrees each), allowing for directional stimulation. Abbott™ (Illinois, United States); DBS: deep brain stimulation

The decision was made to pursue staged bilateral DBS and the STN was selected due to the predominance of akinesia, rigidity, and tremors, as well as the hope for a reduction in levodopa usage. She underwent awake DBS implantation using a stereotactic frame, utilizing direct targeting technique through merging a pre-operative T2 fluid-attenuated inversion recovery (FLAIR) sequence with an intraoperative CT scan. The dorsolateral STN was targeted at the anterior-posterior position of the anterior edge of the red nucleus, aiming for the lateral position of the STN 3 mm lateral to the red nucleus. MER was utilized to identify the dorsal and ventral border of the STN, followed by macrostimulation to confirm a full symptom response without an adverse effect on the left. An Abbott™ (Illinois, United States) eight-contact directional lead was then placed on the left, which allows for stimulation to be delivered in an axially asymmetric fashion (Figure 1).

**Figure 2:**
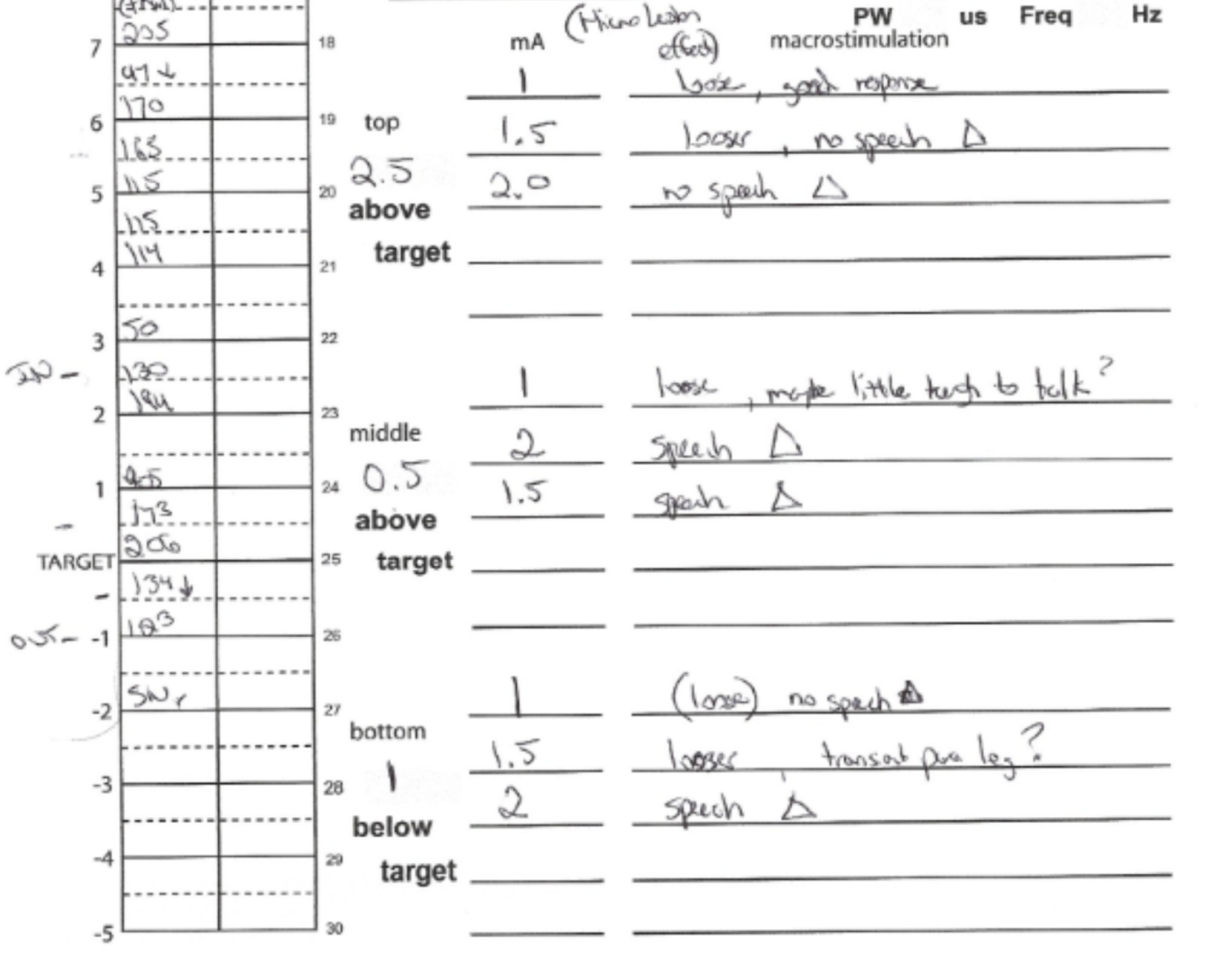
Microelectrode recording and results of macrostimulation of the right-sided lead, showing speech changes at 2 mA 0.5 mm above target and 1 mm below target, suggesting a slightly anteriorly placed lead.

The procedure was then repeated on the right side in a separate session, with macrostimulation producing full rigidity and resting tremor response at 1 mA. Macrostimulation then produced sustained dysarthria at 2 mA 0.5mm above target and 1 mm below the target (Figure 2). This suggested a slightly anteriorly located electrode. Given this finding, the team discussed the option of removing the microelectrode, adjusting the target posteriorly, and then starting a new pass. The patient was becoming anxious with the awake procedure, so it was decided to attempt implanting the permanent Abbott™ eight-contact directional lead to stimulate through the posteriorly oriented electrodes, taking advantage of the directional contact to stimulate away from the anterior side-effects. Intraoperative fluoroscopy was used to confirm accurate lead rotation with the “hourglass” radiographic marker ensuring the A contacts were placed in an anterior direction. This was also done with the knowledge that removal and repeat MER passes could always be subsequently completed if the directional contact failed to impart a large therapeutic window.

Segmented contact 11C was then activated with a monopolar montage intraoperatively, showing full rigidity and tremor response at 0.5 mA with slight facial pulling noted as a side-effect at 4 mA. Given this large therapeutic window (3.5 mA) and full symptom response, the permanent lead was left at this position, sparing the patient the risk and time inherent to repeated microelectrode passes.

Now six months out from lead implantation, she is experiencing continued full tremor and bradykinesia response at 0.55 mA through monopolar stimulation at contact 11C (pulse width 60 µs, rate 160 Hz) and has been able to stop the Rotigotine patch as well as reduce her carbidopa/levodopa/entacapone dose to one tablet of 50/100/200mg 4x daily, a 71.5% reduction in levodopa as compared to preop.

## Discussion

DBS practice patterns, intraoperatively and in programming, have evolved over the last 20 years, limited by the axially symmetric stimulation of the classic leads. But now, the advent of directional DBS has allowed for new options to become part of the classic treatment paradigms. To be able to deliver stimulation in an axially asymmetric fashion gives options where before there were none.

As with this case report, discovering speech-related side-effects at a moderate power would suggest a lead that is slightly anterior, and per the classic treatment paradigm, would then require removal, adjustment posteriorly, and reinsertion to ensure a solid therapeutic window. While DBS is a relatively safe procedure, each pass does impart an increasing and cumulative risk of ICH [7,10-12]. The ability to reduce the number of passes through the use of directional macrostimulation reduces the risk of the procedure by eliminating the need for additional passes.

That being said, this concept is no substitute for an accurately placed lead. In this particular case, the patient’s anxiety with the awake procedure was starting to show, and if the intent of electrode repositioning is to ensure the best symptom response with the largest therapeutic window between symptom control and side-effect, and minimizing the risk of ICH is paramount, then the potential exists to utilize an eight-contact directional DBS lead to fulfill these purposes without needing the time and risk of additional repositioning.

## Conclusions

This index case report describes the intraoperative use of an eight-contact directional deep brain stimulation (DBS) lead to avoid adjustment and repeat microelectrode passes after the initial pass elicited side-effects that suggested a slightly anteriorly placed lead. Limited by the patient's anxiety, the team utilized the directional component of the DBS lead in lieu of minor adjustment and repeat microelectrode passing. Through this approach, a successful result was achieved. This case report suggests that the flexibility of directional stimulation could be considered in the intraoperative setting if axially symmetric macrostimulation would indicate the need for a slight lead adjustment due to a narrow threshold for the onset of a side-effect. This concept should not be considered a substitute for an accurately placed lead.
